# Biometamaterials: Black Ultrathin Gold Film Fabricated on Lotus Leaf

**DOI:** 10.1038/srep15992

**Published:** 2015-11-04

**Authors:** Yuusuke Ebihara, Ryoichi Ota, Takahiro Noriki, Masayuki Shimojo, Kotaro Kajikawa

**Affiliations:** 1 Interdisciplinary Graduate School of Science and Engineering, Tokyo Institute of Technology, Nagatsuta, Yokohama 226-8502, Japan; 2 Department of Materials Science and Engineering, Shibaura Institute of Technology, Koto, Tokyo 135-8548, Japan

## Abstract

We report on a black metamaterial of gold fabricated on a lotus leaf that was used as a template. In spite of the extremely thin gold coating (10-nm thick) on the lotus leaf, the surface shows reflectivity below 0.01 over the entire visible spectral range. Finite-difference time-domain (FDTD) calculations suggest that the low reflectivity stems from the secondary structures on the lotus leaf, where randomly oriented nanorods are distributed.

Lotus is an aquatic plant and its photographic images are shown in Fig. 1(a,b). The surface of a lotus leaf is highly water-repellent. This repellency derives from primary microscopic protuberances with nanoscale secondary roughness composed of wax crystalloids^1^,^2^,^3^. Many researches have focused on the fabrication of artificial super-hydrophobic and self-cleaning surfaces by mimicking the surface structure of lotus leaves^4^,^5^,^6^,^7^,^8^,^9^,^10^.

Magnified scanning electron microscopy (SEM) images revealed that the secondary roughness is due to assemblies of randomly oriented nanorods, as shown in Fig. 1(c). Such a structure may be a good template or component for metamaterials to confine light, since good light-absorbing properties of vertically oriented nanorods have been reported in the literature, although the dimensions and material differ from those in our study^11^,^12^. The importance of optical absorbers over a broad spectral range has been recognized and some works have been carried out on this topic^11^,^12^,^13^,^14^. In this letter, we report on a black metamaterial formed on a lotus leaf (*Nelumbo nucifera*). Although the gold coating on the leaf is extremely thin (10-nm thick), the surface is very black with a reflectivity below 0.01 over the entire visible spectral range.

The samples were prepared by the following procedure: (i) Lotus seeds were sowed in water with very small amount of liquid fertilizer. (ii) After a few weeks, the cotyledon was nipped off and fixed on a glass slide. (iii) A thin gold film was deposited on the leaves. The gold coating was carried out using two methods: sputtering in air at low pressure and vacuum evaporation using resistant thermal heating. The sputtering was performed using an E-1030 sputtering coater (Hitachi), and the vacuum evaporation was carried out in a VE-2030 evaporator (Shinku Device). For control, we prepared the samples of gold-sputtered leaves of a Japanese pepper tree (*Zanthoxylum piperitum*), dokudami (*Houttuynia cordata*) and mugwort (*Artemisia indica*). Their photographs are given in Figure S1 in Supporting Information.

## Experimental

The reflection spectra were recorded with a MCPD-3000 spectrometer (Otsuka Electronics) using a halogen lamp as a light source. For the reflectivity measurements, the light was conveyed to the sample with a Y-type optical fiber (400 *μ*m in diameter) and the reflected light was collected by it. The light was irradiated at normal incidence. For the scattering measurements, the light from the halogen lamp was likewise conveyed by an optical fiber to the sample at normal incidence. The back-scattered light was collected by another optical fiber and transferred to the spectrometer. As a reference, an SRS-99 diffuse reflectance standard (Labsphere) was used. The scattering angle was approximately 60° with respect to the surface normal. SEM observations were performed with an S-4500 SEM (Hitachi).

## Results and Discussion

The photographic images of gold-coated and uncoated lotus leaves are shown in Fig. 2(a–e). These samples are designated as samples I–V, respectively. The gold coating was done by sputtering (samples I–III) and vacuum evaporation using resistant thermal heating (sample IV). An untreated lotus leaf is shown for reference (sample V). The thicknesses of the gold coatings are (a) 10 nm, (b) 20 nm, (c) 30 nm and (d) 30 nm. The surface of the lotus leaf is fixed by polyimide or carbon tape, which looks lustrous in the images Fig. 2(a–c, f). These images show that the surfaces of samples I–III are black while the color of sample IV is gold. This difference is due to the deposition method used, as shown later. For control, we sputtered gold on the leaves of a Japanese pepper tree, dokudami and mugwort (*Artemisia indica*), at a thickness of 30 nm. They are metallic and gold-colored like sample IV, as shown in Fig. 2(f) (dokudami leaf), in contrast to the black lotus leaves covered with gold. The photographic images of other leaves are shown in Figure S2 in Supporting Information. Therefore, the black surface is produced only in the lotus leaves.

**Figure 1:**
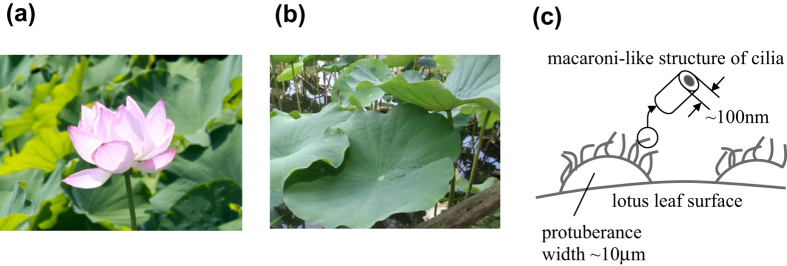
(**a**) A flower and (**b**) leaves of lotus. (**c**) A model of surface nanostructure of a lotus leaf.

Figure 3(a–d) show coarse SEM images of samples I–IV. Samples I–III have microscopic protuberances of size of about 10–20 *μ*m. The protuberances are surrounded by secondary structures of cilia^1^, as illustrated in Fig. 1(c). In sample IV, the protuberances collapsed and their surface seems to be smooth (Fig. 3(d)). Figure 3(e–h) show the magnified SEM images of the surfaces of the protuberances of samples I–IV at 10-times higher magnification. The SEM images of the lotus leaf covered with sputtered gold reveal many macaroni-like nanorods that are randomly oriented. The outer diameter of these nanorods is approximately 100 nm and they are hollowed out with an inner diameter of approximately 50 nm. In Fig. 3(h), secondary structures are absent and the surface of the lotus leaf is rather flat, apart from cracks. Vacuum-evaporation of gold using resistant thermal heating apparently results in the destruction of the secondary nanostructures. The photographic image of sample IV showing its golden color results from the absence of the secondary nanostructures. The SEM images of other leaves are shown in Figure S2 in Supporting Information.

**Figure 2:**
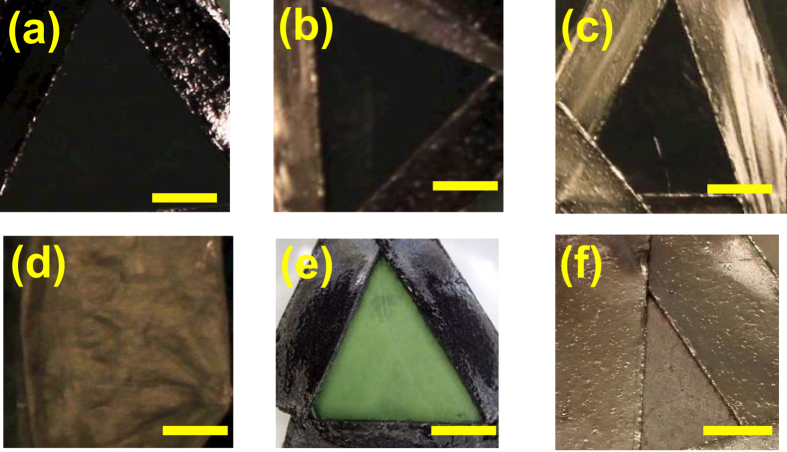
Photographs of the samples (bar: 5 mm): lotus leaves covered with a sputtered gold film (**a**) 10-nm, (**b**) 20 nm and (**c**) 30-nm-thick (sample I–III), (**d**) a lotus leaf covered with 30-nm-thick vacuum-evaporated gold (sample IV), (**e**) lotus leaf without coating (sample V), and (**f**) a dokudami leaf covered with a 30-nm-thick sputtered gold film (sample VI) for control.

**Figure 3:**
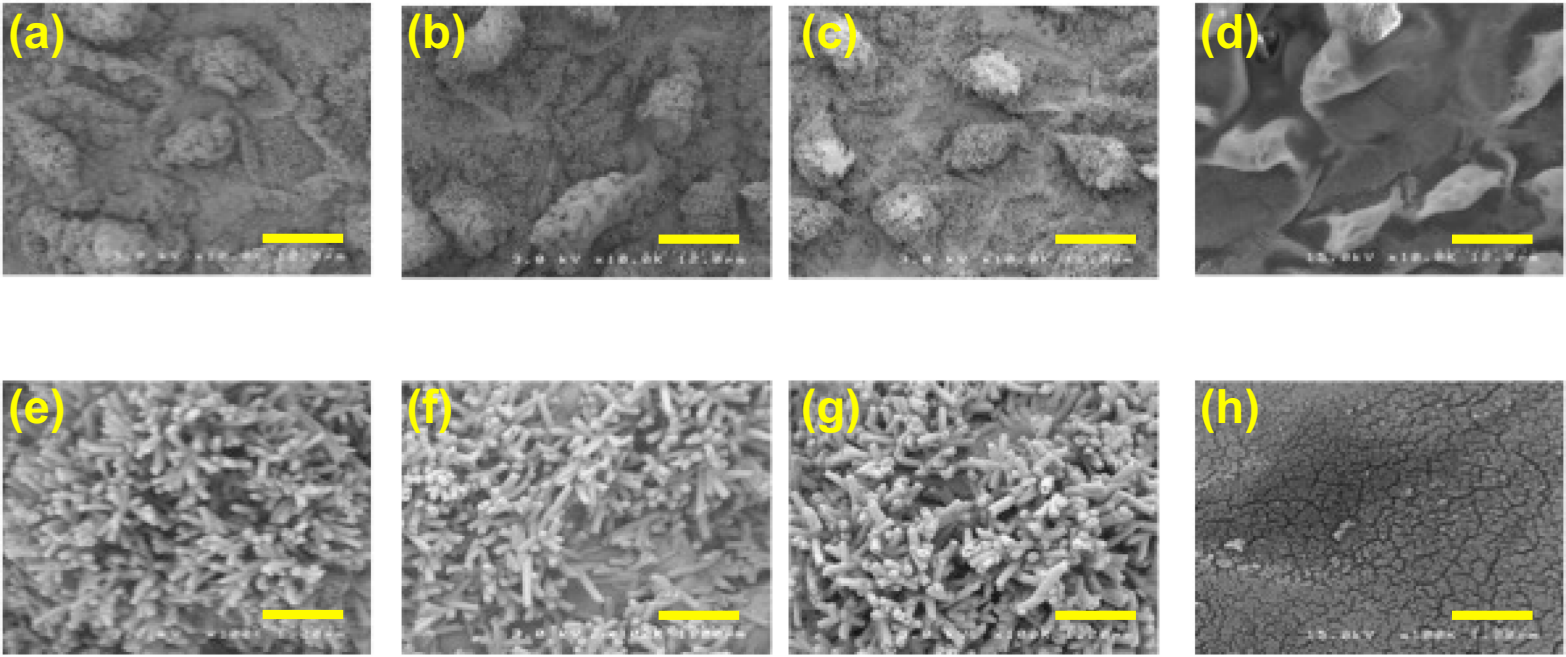
Upper row: Coarse SEM images of (**a**) sample I, (**b**) sample II, (**c**) sample III, and (**d**) sample IV. (bar: 10 *μ*m) Lower row: Magnified images of (**e**) sample I, (**f**) sample II, (**g**) sample III, and (**h**) sample IV. (bar: 1 *μ*m).

**Figure 4:**
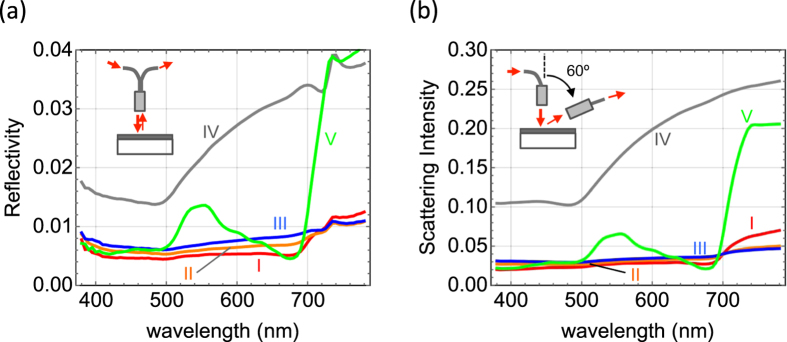
(**a**) Reflection spectra of samples I–V measured at normal incidence. (**b**) Measured scattering spectra of samples I–V. The scattering intensity is normalized by that from the reference.

Figure 4(a,b) show the reflection and scattering spectra, respectively. The scattering intensity is normalized by that from the reference (SRS-99 diffuse reflectance standard). The reflectivity of samples I–III is lower than 0.01 over the entire visible spectral range, whereas sample IV has a reflectivity of a few percent. The low reflectivity of samples I–III is consistent with the images shown in Fig. 2(a–c), in which the surface appears black. The scattering spectra shown in Fig. 4(b) are similar to the reflection spectra. Concerning sample V, the scattering and reflection spectra show a peak at 550 nm and it is considerably higher at long wavelengths (700–800 nm), whereas scattering and reflection is very weak at wavelengths of 400–500 nm and 600–700 nm. The weak scattering is due to strong absorption of chlorophyll in the leaf at visible wavelengths^15^.

The low reflectance over the entire visible spectral range in samples I–III is due to the gold coating on the lotus leaf. This is supported by the fact that the reflectivity is independent of the thickness of the gold coating and that it stays low at long wavelengths (700–800 nm), in spite of strong reflection and scattering at wavelengths of 700–800 nm. The slightly higher reflectivity and scattering intensity in sample I at this wavelength regime is due to the thin gold coating. The low reflectance of gold coating over the visible range also implies that other metals such as silver can be used for coating, because silver is more metallic with lower loss. For the same reason, the low reflectance will be hold at the wavelengths longer than 800 nm where gold has lower loss.

**Figure 5:**
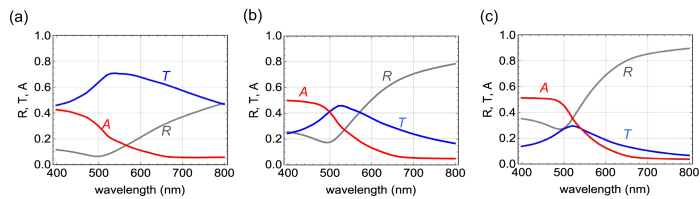
Calculated reflectivity *R*, transmittance *T*, and absorption *A* spectra of (**a**) 10-nm-, (**b**) 20-nm-, and (**c**) 30-nm-thick flat gold films.

Figure 5(a–c) show the calculated reflectivity *R*, transmittance *T*, and absorption *A* of gold films that are 10-, 20-, and 30-nm thick, respectively. The absorption *A* is evaluated using the relation 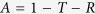, because no scattering takes place in flat gold films. The calculation was done using the transfer-matrix method^16^ with refractive indices of gold taken from the literature^17^. The reflectivity *R* is more than 0.1 over the entire visible spectral range even in case of the 10-nm-thick gold film, and it is more than 0.3 for the 30-nm-thick gold film. Although the profiles of *T* and *R* vary with the thickness of the film, *A* is almost independent of thickness. While *A* ~ 0.5 in the wavelength range of 400–500 nm, the absorption is weak 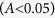 at longer wavelengths (650–800 nm). The reflectivity observed in samples I–III is much lower than that of the flat gold film that is 10-nm thick, indicating that the lotus leaf with ultrathin gold coating has considerably low reflectivity. This low reflectivity stems from the metallic surface structure, where the incident light is confined and hardly emitted. A similar process is reported in vertically aligned single-walled carbon nanotubes, which show excellent blackbody properties over a wide spectral range^11^. In addition, great light absorption has recently been reported in sharply convex gold structures^12^.

**Figure 6:**
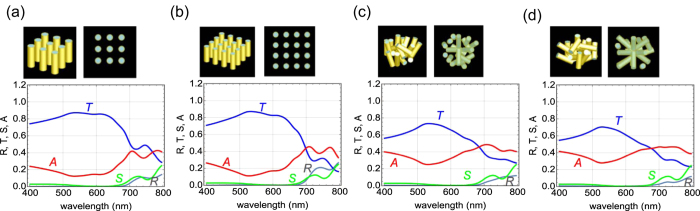
Lower row, main frames: FDTD-calculated reflectivity *R*, transmittance *T*, scattering *S*, and absorption *A* spectra of (**a**) model A, (**b**) model B, (**c**) model C, and (**d**) model D. Upper row, small frames: Perspective (left) and top view (right) of each model. The diameter of each nanorod-core is 100 nm. Each nanorod is coated with 10-nm-thick gold film. The length of the nanorods is 380 nm.

The importance of such randomly orientated structures to produce a black metamaterial is confirmed by finite-difference time-domain (FDTD) calculations. The calculations were performed using FDTD Solutions (Lumirical Solutions Inc.). Models A and B consist of 9 and 16 nanorods (100-nm diameter and 380-nm long) covered with 10-nm-thick gold film, respectively. Perspective and top views are shown in the upper row of Fig. 6. The refractive index of the nanorods is 1.48 and the refractive index of gold is taken from the literature^17^. The nanorods are aligned in a two-dimensional square lattice with 100 nm spacing between the nanorods, as shown in Fig. 6(a,b). Models C and D consist of randomly oriented nanorods and their perspective and top views are shown in Fig. 6(c,d). In the two randomly oriented models, the number of nanorods is 17, similar to that of model B (16). The volume of gold of each nanorod is 1.313 × 10^−3^ *μ*m^3^. The amount of gold contained in the 16 nanorods is almost equal to that of the 20-nm-thick gold film with an area of 1 *μ*m^2^ (2 × 10^−2^ *μ*m^3^).

The reflectivity (back scattering) *R*, transmittance (front scattering) *T*, and scattering (side scattering) *S* are calculated using total-field scattered-field sources for illumination. The investigated structure includes the calculation space of 1 *μ*m × 1 *μ*m × 1 *μ*m, surrounded by perfectly matched layers. The mesh size was 2 nm. The absorbance *A* is evaluated by the relation 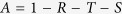. The calculated results of models A–D are shown in the main frames of Fig. 6(a–d), respectively. The reflectivity is below 0.02 in all models at wavelengths shorter than 700 nm. The low reflectivity over the wavelength range of the models is consistent with the experimental results shown in Fig. 4, indicating that the nanorod structures strongly reduce reflection over the entire visible spectral range. At longer wavelengths, *R* stays low (less than 0.15) in models C and D, while it is higher than 0.2 in models A and B, indicating that the random structure reduces the reflectivity. The absorbance profiles of models C and D are much greater than those of the other models and that of the flat gold films shown in Fig. 5, although the amount of gold is almost the same in models B–D and the 20-nm-thick flat gold film. This means that randomly oriented metallic nanorod structures are good light absorbers. Lotus leaves provides us with a good template for such a black metamaterial structure.

## Conclusion

We fabricated a black metamaterial using a lotus leaf as a template. Despite the fact that the gold-film coating was very thin (10-nm thick), extremely low reflectance of less than 0.01 was observed over the entire visible spectral range. This low reflectance and scattering originates from the macaroni-like nanorods on the lotus leaves. The low reflectance was confirmed by FDTD calculations using a model consisting of randomly oriented gold-coated nanorods. Although it is difficult to remove the black metamaterial mechanically from the template, it may be removed by chemical treatment with a base such as sodium hydroxide. This work is in process and will be reported elsewhere.

## Additional Information

**How to cite this article**: Ebihara, Y. *et al.* Biometamaterials: Black Ultrathin Gold Film Fabricated on Lotus Leaf. *Sci. Rep.* **5**, 15992; doi: 10.1038/srep15992 (2015).

## Supplementary Material

Supplementary Information
