# Supplementary material for: Biometamaterials: Black Ultrathin Gold Film Fabricated on Lotus Leaf
Source: Sci Rep. 2015 Nov 4;5:15992. doi: 10.1038/srep15992 (PMC4632130; doi:10.1038/srep15992)
Supplement: Supplementary Information [file srep15992-s1.pdf]

Supporting Information

Biometamaterials: Black Ultrathin Gold Film Fabricated on Lotus Leaf

Yuusuke Ebihara,[1] Ryoichi Ota,[2] Takahiro Noriki,[2] Masayuki Shimojo[2] and Kotaro Kajiakwa[1]

[1]Interdisciplinary Graduate School of Science and Engineering, Tokyo Institute of Technology, Nagatsuta, Yokohama 226-8502, Japan

[2]Department of Materials Science and Engineering, Shibaura Institute of Technology, Koto, Tokyo 135-8548, Japan

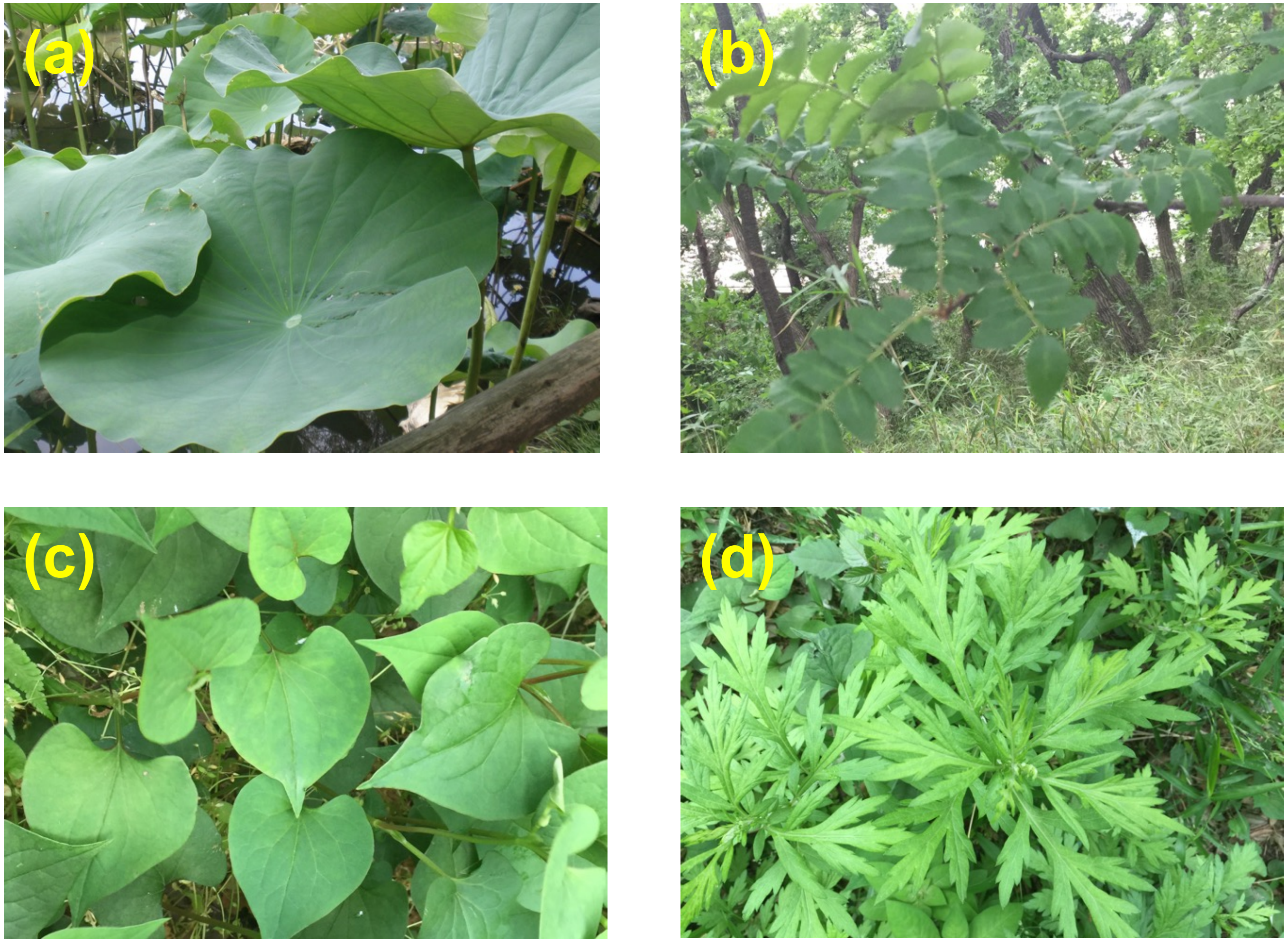

Figure S1
Photographic images of (a) lotus, (b) Japanese pepper tree, (c) dokudami and (d) mugwort.

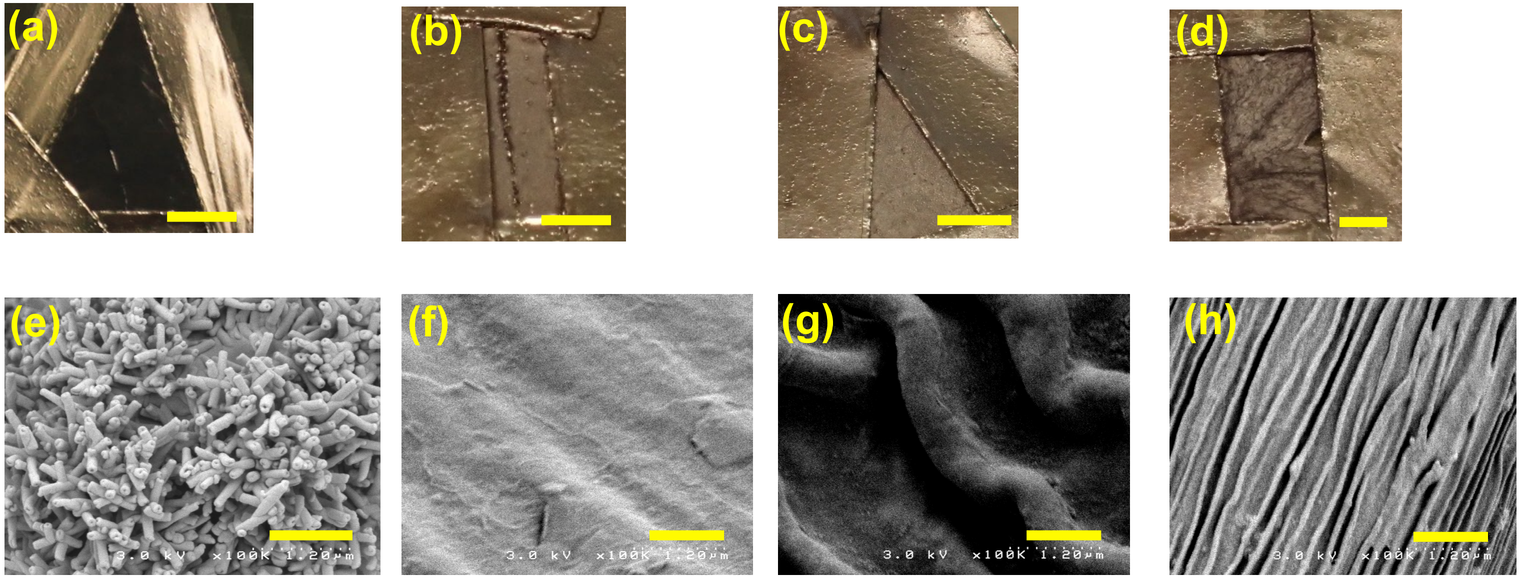

Figure S2

Photographic images of the leaves covered with gold (a) lotus, (b) Japanese pepper tree, (c) dokudami and (d) mugwort. The bar is 5 mm. SEM images of (e) lotus, (f) Japanese pepper tree, (g) dokudami and (h) mugwort. The bar is 1 μm.
